# Diet-Induced Obesity Impairs Endothelium-Derived Hyperpolarization via Altered Potassium Channel Signaling Mechanisms

**DOI:** 10.1371/journal.pone.0016423

**Published:** 2011-01-21

**Authors:** Rebecca E. Haddock, T. Hilton Grayson, Margaret J. Morris, Lauren Howitt, Preet S. Chadha, Shaun L. Sandow

**Affiliations:** 1 Department of Pharmacology, School of Medical Sciences, University of New South Wales, Sydney, New South Wales, Australia; 2 Department of Neuroscience, John Curtin School of Medical Research, Australian National University, Canberra, Australian Capital Territory, Australia; University of Illinois at Chicago, United States

## Abstract

**Background:**

The vascular endothelium plays a critical role in the control of blood flow. Altered endothelium-mediated vasodilator and vasoconstrictor mechanisms underlie key aspects of cardiovascular disease, including those in obesity. Whilst the mechanism of nitric oxide (NO)-mediated vasodilation has been extensively studied in obesity, little is known about the impact of obesity on vasodilation to the endothelium-derived hyperpolarization (EDH) mechanism; which predominates in smaller resistance vessels and is characterized in this study.

**Methodology/Principal Findings:**

Membrane potential, vessel diameter and luminal pressure were recorded in 4^th^ order mesenteric arteries with pressure-induced myogenic tone, in control and diet-induced obese rats. Obesity, reflecting that of human dietary etiology, was induced with a cafeteria-style diet (∼30 kJ, fat) over 16–20 weeks. Age and sexed matched controls received standard chow (∼12 kJ, fat). Channel protein distribution, expression and vessel morphology were determined using immunohistochemistry, Western blotting and ultrastructural techniques. In control and obese rat vessels, acetylcholine-mediated EDH was abolished by small and intermediate conductance calcium-activated potassium channel (SK_Ca_/IK_Ca_) inhibition; with such activity being impaired in obesity. SK_Ca_-IK_Ca_ activation with cyclohexyl-[2-(3,5-dimethyl-pyrazol-1-yl)-6-methyl-pyrimidin-4-yl]-amine (CyPPA) and 1-ethyl-2-benzimidazolinone (1-EBIO), respectively, hyperpolarized and relaxed vessels from control and obese rats. IK_Ca_-mediated EDH contribution was increased in obesity, and associated with altered IK_Ca_ distribution and elevated expression. In contrast, the SK_Ca_-dependent-EDH component was reduced in obesity. Inward-rectifying potassium channel (K_ir_) and Na^+^/K^+^-ATPase inhibition by barium/ouabain, respectively, attenuated and abolished EDH in arteries from control and obese rats, respectively; reflecting differential K_ir_ expression and distribution. Although changes in medial properties occurred, obesity had no effect on myoendothelial gap junction density.

**Conclusion/Significance:**

In obese rats, vasodilation to EDH is impaired due to changes in the underlying potassium channel signaling mechanisms. Whilst myoendothelial gap junction density is unchanged in arteries of obese compared to control, increased IK_Ca_ and Na^+^/K^+^-ATPase, and decreased K_ir_ underlie changes in the EDH mechanism.

## Introduction

Obesity is at epidemic levels, with cardiovascular dysfunction being a common outcome [1]. Obesity is associated with an increased incidence of type-2 diabetes, hypertension, stroke, metabolic syndrome, peripheral arterial disease and myocardial infarction, and thus makes a significant contribution to premature death [1]. However, the mechanisms of vascular dysfunction in obesity are poorly understood.

Vascular tone refers to the balance between constrictor and dilator influences and is critical for the control of blood flow and pressure, and thus for normal cardiovascular function. The endothelium is a major regulator of vascular tone, producing vasoconstrictor agents such as metabolites of arachidonic acid, superoxide anions, angiotensin II and endothelin-1 [2], and vasodilator action due to nitric oxide (NO), cyclooxygenase and a NO/cyclooxygenase-independent endothelium-derived hyperpolarization (EDH) mechanism [3], [4], [5]. Characterization of the EDH response in health and disease is critical, as such activity generally underlies the primary vasodilator mechanism in the smaller resistance vessels that are integral for control of vascular tone and blood flow [4], [6], [7].

The EDH response is well characterized in rat mesenteric artery. In this vessel, agonist-induced EDH is dependent on inositol 1,4,5-trisphosphate (IP_3_)-mediated release of intracellular calcium [5], [8], [9] and subsequent endothelial small (S) and intermediate (I) conductance calcium-activated potassium channel (K_Ca_) activation [3], [5]. In turn, these channels release K^+^ into the localized myoendothelial space, and/or facilitate the initiation of a hyperpolarizing current which is transferred to smooth muscle via myoendothelial gap junction connexins (Cxs [10], [11], [12]). Hyperpolarization is also initiated via K^+^ in the localized myoendothelial space activating smooth muscle Na^+^/K^+^ATPase, whilst endothelial cell inward rectifying potassium channels (K_ir_) may be involved in amplifying the response [13]. The net smooth muscle hyperpolarization facilitates closure of voltage-dependent calcium channels to initiate vessel relaxation [3], [5]. In healthy normal adult rat mesenteric artery, a significant proportion of potassium and Cx-mediated signaling, which are essential for EDH, occurs at localized myoendothelial microdomain sites [5], [10], [11], [12], [13].

Vascular disease associated with endothelial dysfunction includes type-2 diabetes and hypertension (for review [6]) linked to obesity. Indeed, EDH is impaired in mesenteric artery of insulin-resistant type-2 diabetic obese Zucker rats, via altered K_Ca_ [14] and gap junction Cx-dependent [15] EDH mechanisms. Further, in skeletal muscle saphenous artery branches of the diet-induced obese rat, myoendothelial microdomain gap junctions and IK_Ca_ are upregulated and account for EDH activity [16].

In the present study, the ability of 4^th^ order mesenteric arteries to develop myogenic tone provides a physiologically relevant framework from which to examine vasodilation. Indeed, mesenteric arteries from control and diet-induced obese rats develop myogenic tone at physiologically relevant pressure (80 mmHg; [17]). This is in contrast to altered myogenic tone in skeletal and renal arterioles of the Zucker rat [18], [19], but similar to observations in diet-induced obese rat gracilis muscle and coronary arterioles [20], [21], as well as human forearm vessels from obese individuals [22]; suggesting that the genetic Zucker obesity model does not generally reflect the diet-induced obese state in humans. Further, in contrast to the leptin receptor-deficient Zucker rat, where obesity develops independently of circulating leptin [23], the diet-induced obese rat directly reflects common forms of human obesity, where leptin, insulin, glucose, triglycerides and blood pressure are elevated [1], [23], [24], [25], [26].

The present study aimed to examine the effect of diet-induced obesity on EDH-mediated function and the underlying mechanisms in rat mesenteric artery with myogenic tone.

## Results

### General features of diet-induced obese rats

The effect of dietary intervention on biochemical and metabolic parameters was characterized in control and obese rats. Following the 16–20 week diet intervention, animals on the obese diet were ∼37% heavier than age-matched controls (*P*<0.05; Table 1). Body length, kidney and liver weight, retroperitoneal and gonadal fat mass, blood glucose, insulin and leptin levels of obese animals were significantly elevated (*P*<0.05) compared to controls (Table 1).

**Table 1:** Control and diet-induced obese rat characteristics.

	Control (*n = *65)	Obese (*n = *45)
Body weight (g)	530±6	723±15*
Body length (cm)	25.5±0.1	26.7±0.1*
Blood glucose (mM)	8.1±0.1	9.7±0.3*
Insulin (ng/ml)	4.5±0.5	12.6±0.5*
Leptin (ng/ml)	5.3±0.2	9.1±0.2*
Kidney (g)	1.5±0.1	1.8±0.1*
Liver (g)	17.7±0.3	24.5±0.8*
Retroperitoneal fat (g)	6.2±0.2	21.8±1.1*
Gonadal fat (g)	8.8±0.3	24.7±1.0*

*, *P<*0.05, compared to control.

### Effect of obesity on general anatomical vessel characteristics

**Figure 1:**
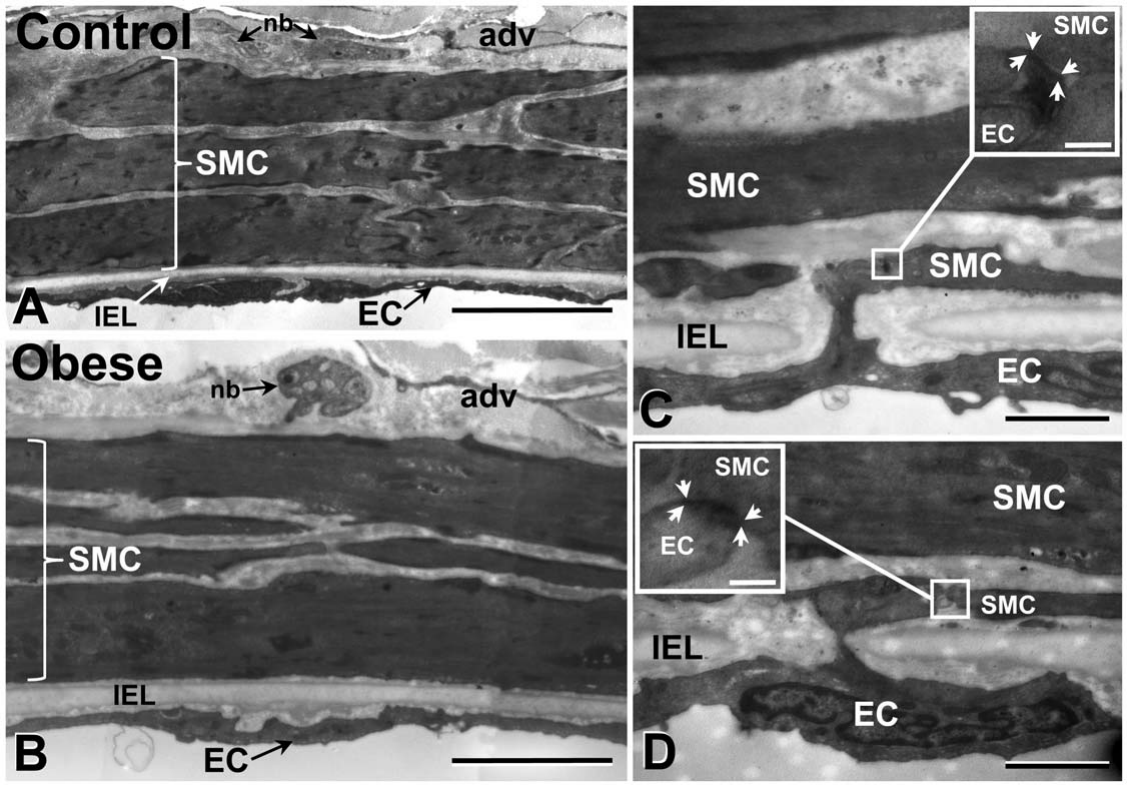
Morphology of control and obese rat mesenteric artery. The media of obese rat mesenteric arteries contain more smooth muscle cell (SMC) layers than control (**A, B;** **Table 2**). Myoendothelial gap junctions were present on endothelial cell (EC) projections in both control and obese arteries (**C, D** and insets). Such sites have characteristic pentalaminar gap junction morphology (inset, between arrows). adv, adventitia; IEL, internal elastic lamina; nb, nerve bundle. Bars; **A, B**, 10 µm; **C, D**, 1 µm; inset, 100 nm.

The effect of dietary intervention on mesenteric artery morphology was characterized in control and obese rats. Mesenteric arteries from obese rats had a significant increase in medial smooth muscle cell layers, medial thickness and media to lumen ratio compared to control (*P*<0.05; Table 2, Figs. 1, S1). No differences were observed in vessel diameter or medial cross sectional area (Table 2). Myoendothelial gap junctions were identified in both control and obese arteries and were present at the same density and proportion of IEL holes in arteries of control and obese rats (Table S1, Fig. 1).

**Table 2:** Control and diet-induced obese rat mesenteric artery characteristics.

	Diameter(µm)	# SMC layers	Medial thickness (µm)	Medial CSA(µm^2^)	Media to lumen ratio (×10^**−2**^)
Control	199±21	2.9±0.2	10.4±0.4	3303±323	4.90±0.27
Obese	221±12	3.9±0.3*	12.9±0.6*	3877±238	6.18±0.26*

*n = *8, each from a different rat.

*, *P*<0.05, compared to control. CSA, cross-sectional area; SMC, smooth muscle cell.

### Effect of obesity on the myogenic response

**Figure 2:**
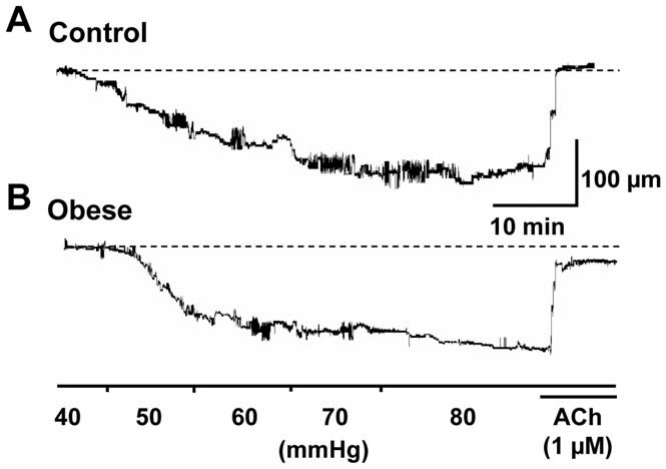
Myogenic response. Representative traces of myogenic tone development in arteries from control (**A**) and obese (**B**) animals with stepped pressure increases. At 80 mmHg, no difference was observed in the degree of constriction in control compared to obese arteries, with ACh (1 µM) relaxing arteries from control and obese rats.

**Figure 3:**
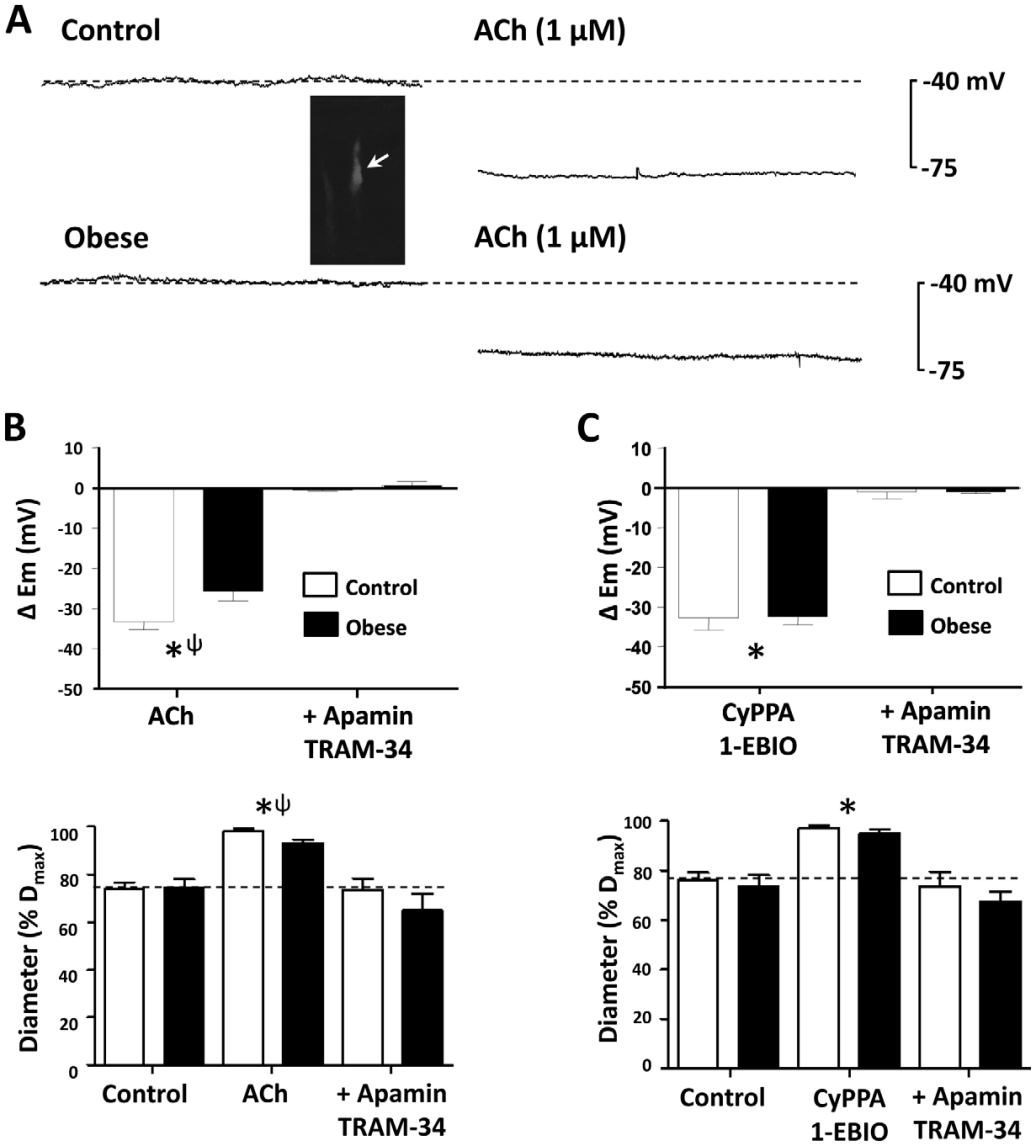
Combination SK_Ca_ and IK_Ca_-mediated function. Representative membrane potential recordings show impaired hyperpolarization to ACh (1 µM) in pressurized obese, compared to control rat mesenteric artery (**A**, inset; impaled propidium iodide identified muscle cell; longitudinal vessel axis, *left* to *right*; bar, 10 µm). Hyperpolarization and relaxation to ACh were abolished by apamin (50 nM) and TRAM-34 (1 µM), confirming endothelium-derived hyperpolarization patency (**B**; Table S3). Hyperpolarization and relaxation could also be induced by the SK_Ca_ and IK_Ca_ agonists CyPPA (30 µM) and 1-EBIO (300 µM), with subsequent block by apamin and TRAM-34 (**C**; Table S3). Arteries from obese animals were further associated with a constriction beyond resting tone (**B, C**). *P<0.05 from own control; ψ, *P*<0.05 control compared to obese. ΔE_m_, change in membrane potential from own control.

The effect of dietary intervention on the myogenic response was characterized in mesenteric artery of control and obese rats. Arteries from both control and obese rats routinely developed myogenic tone (Fig. 2A, B), although there was no difference in the degree of constriction at 80 mmHg between diet groups (Table S2). The D_max_ in 0 mM Ca^2+^ physiological salt solution was not significantly different in arteries from control and obese rats (Table S2). Intracellular recordings from smooth muscle cells showed that resting membrane potential at 80 mmHg was also not significantly different between the two groups (Fig. 3A; Table S3).

### Mesenteric artery EDH

5w?>The effect of dietary intervention on the characteristics of EDH was characterized in mesenteric artery of control and obese rats. In the presence of *N*_ω_-Nitro-L-arginine methyl ester hydrochloride (L-NAME; 1 µM), 1H-[1], [2], [4]oxadiazolo[4,3-a]quinoxalin-1-one (ODQ; 100 µM) and indomethacin (10 µM), acetylcholine (ACh; 1 µM) produced a hyperpolarization and relaxation, which was impaired in arteries from obese animals (*P<*0.05, Fig. 3A, B). In arteries from both diet groups, combined apamin (50 nM) and 1-[(2-chlorophenyl)diphenyl-methyl]-1H pyrazole (TRAM-34; 1 µM), blockers of SK_Ca_ and IK_Ca_ respectively, abolished ACh-induced hyperpolarization and relaxation (*P*<0.05, Fig. 3B), while apamin and TRAM-34, in the absence of ACh, had no significant effects on vessel diameter (Table S2). Of note, in 4^th^ order mesenteric arteries, the ACh response in the presence of L-NAME, ODQ and indomethacin was not different compared with EDH associated relaxation evoked by ACh in the absence of these blockers (Table S2), consistent with the primary contribution of EDH to endothelium-dependent relaxation in such small distal mesenteric vessels [27].

Hyperpolarization and relaxation of mesenteric artery smooth muscle could also be evoked by a combination of the concentration selective SK_Ca_ and IK_Ca_ agonists cyclohexyl-[2-(3,5-dimethyl-pyrazol-1-yl)-6-methyl-pyrimidin-4-yl]-amine (CyPPA; 30 µM [16], [28], [29]) and 1-ethyl-2-benzimidazolinone (1-EBIO; 300 µM [16], [30]) in arteries from control and obese rats (*P<*0.05, Fig. 3C). The magnitude of this response was similar to that recorded in the presence of ACh (Fig. 3B). Likewise, apamin and TRAM-34 in combination, blocked the CyPPA/1-EBIO evoked hyperpolarization and relaxation (*P<*0.05, Fig. 3C).

### IK_Ca_-dependent EDH is upregulated in obese rat mesenteric artery

**Figure 4:**
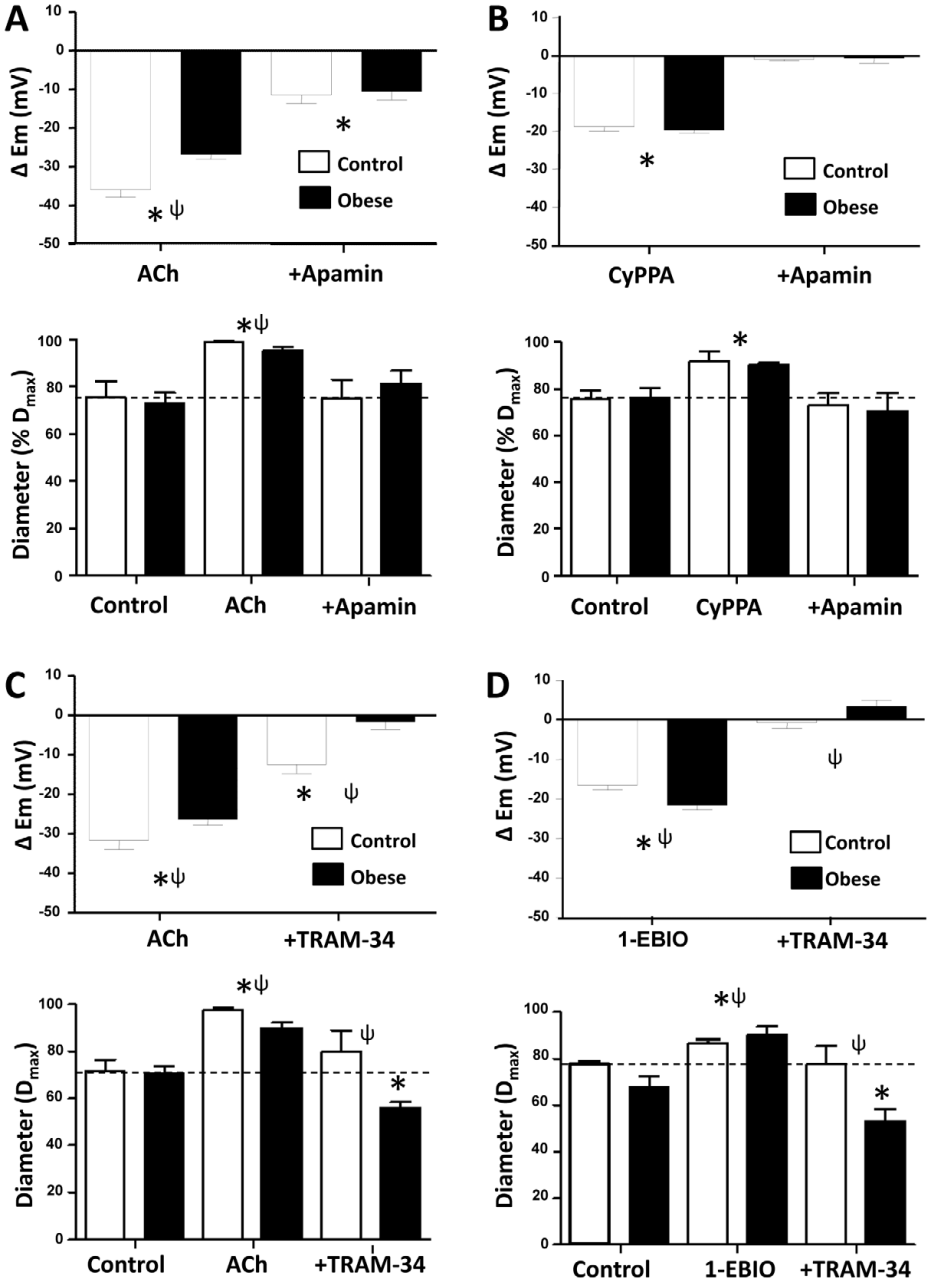
Selective SK_Ca_ and IK_Ca_-mediated function. Inhibition of SK_Ca_ by apamin (50 nM) attenuates ACh (1 µM)-induced hyperpolarization in arteries from control and obese rats, and abolished the associated relaxation in control (**A**; Tables S2 and S3). Hyperpolarization and relaxation elicited by CyPPA (SK_Ca_ agonist; 30 µM) is abolished by apamin (**B**; Tables S2 and S3). EDH activity was abolished in arteries from obese rats following inhibition of IK_Ca,_ and was reduced in arteries from control rats (**C**; Table S3). Hyperpolarization and relaxation elicited by 1-EBIO (300 µM; activating IK_Ca_) was larger in arteries from obese compared to control, with both being inhibited by TRAM-34 (**D**; Tables S2 and S3). *P<0.05 from own control; ψ, *P*<0.05 control compared to obese. ΔE_m_, change in membrane potential from control.

The effect of dietary intervention on the selective characteristics of SK_Ca_ and IK_Ca_-dependent EDH was characterized in mesenteric artery of control and obese rats. The role of SK_Ca_ in the mesenteric artery EDH response was investigated using apamin and CyPPA (Fig. 4A, B). Inhibition of SK_Ca_ with apamin reduced ACh-induced EDH in arteries from both control and obese rats (*P<*0.05; Fig. 4A), although SK_Ca_ contribution to EDH was greater in arteries from control (*P<*0.05; Table S3). Further, direct activation of SK_Ca_ by CyPPA evoked hyperpolarization and relaxation which was not different between arteries from control and obese rats (*P*>0.05; Fig. 4B); this response being blocked by apamin (*P<*0.05), and returning to normal resting conditions in arteries from control and obese animals.

**Figure 5:**
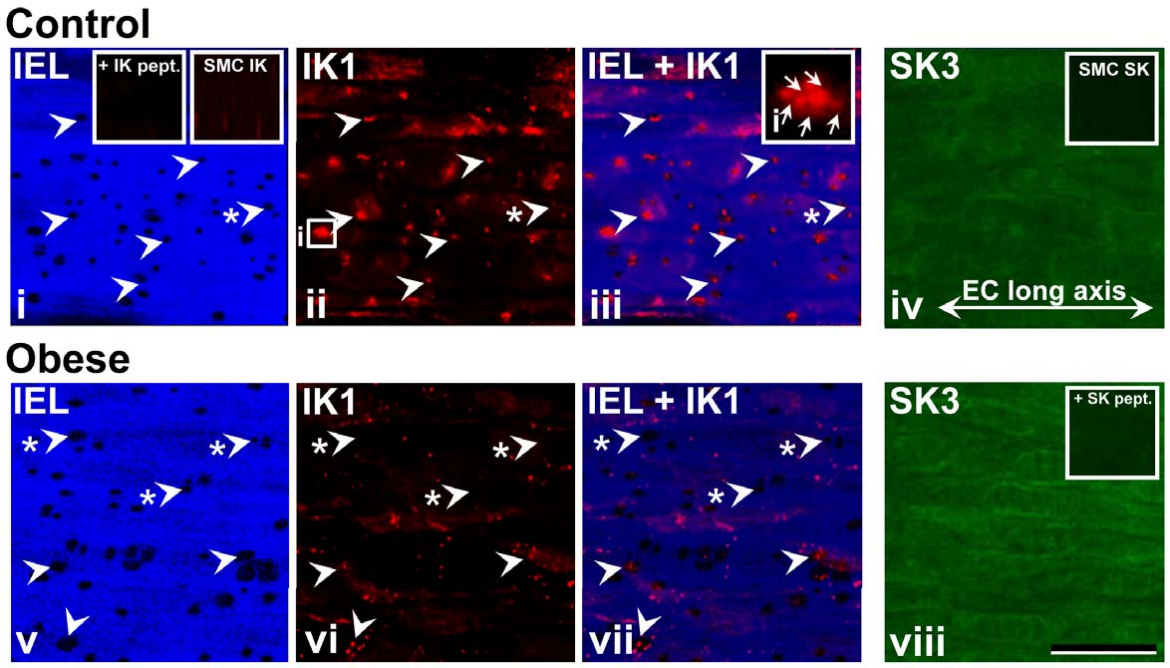
SK3 and IK1 distribution. Confocal immunohistochemisty demonstrates SK3 (SK_Ca_) and IK1 (IK_Ca_) distribution in arteries from control(**i–iv**) and obese (**v–viii**) rat (NB. same vessel region shown in **i–iii** and **v–vii**, respectively). Autofluorescence demonstrates internal elastic lamina (IEL) morphology and associated holes (**i–iii**, and **v–vii**, examples arrowed) as potential myoendothelial gap junction/microdomain sites. IK_Ca_ labeling shows intense punctate localization at discrete points in the endothelium of arteries from control and obese rats (**i–iii** and **v–vii**, respectively), which correlates to IEL hole sites (examples with arrows; see **Table S1**) to a greater extent in obese (**v–vii**) compared to control (**i–iii**; examples of IEL holes without IK_Ca_ localization arrowed, asterisk; see **Table S1**). Aggregates of IK_Ca_ densites of >1 plaque occur in control and obese arteries (example, boxed region in ii shown in iii, inset). Reconstructed confocal series of the full endothelial depth show diffuse SK_Ca_ labeling across the cell surface and cytoplasm of control (**iv**) and obese (**viii**) rat arteries, and lack of localization to IEL hole sites. IK_Ca_ and SK_Ca_ were absent in smooth muscle (**i**, right inset; **iv**, inset). Controls, as peptide block (**i**, left inset and viii, inset, respectively) and incubation in secondary antibody alone (data not shown) show absence of labeling. In all panels, longitudinal vessel axis runs left to right (example, **iv**). Bar, 20 µm; inset box width, 5 µm.

In arteries from control animals, IK_Ca_ inhibition with TRAM-34 reduced ACh-mediated hyperpolarization and the associated relaxation (*P<*0.05; Fig. 4C). However, hyperpolarization and relaxation were abolished by TRAM-34 in mesenteric arteries from obese rats (Fig. 4C). IK_Ca_ activation by 1-EBIO caused a smooth muscle hyperpolarization that was larger in arteries from obese compared to control animals (*P<*0.05; Fig. 4D). In this series of experiments, the larger hyperpolarization evoked by 1-EBIO in arteries from obese animals was associated with increased relaxation (*P<*0.05; Fig. 5D). TRAM-34 abolished the 1-EBIO induced hyperpolarization and relaxation (*n = *4, *P<*0.05, Fig. 4D).

### SK_Ca_ and IK_Ca_ protein in obesity

In order to determine whether the changes in SK_Ca_ and IK_Ca_ activity in diet-induced obese rat mesenteric artery reflect altered IK_Ca_ protein distribution, characterized antibodies were used with confocal immunohistochemistry and, for IK_Ca_ only, Western blotting, on whole arteries.

SK3 (SK_Ca_) and IK1 (IK_Ca_) distribution was determined using confocal immunohistochemistry (Fig. 5). When series of images were taken across the full depth of the vessel wall, reconstruction revealed the presence of SK3 labelling in both control and obese rat arteries as diffuse localization across the cell surface and the cytoplasm of endothelial, but not smooth muscle cells, with such labeling not being associated with IEL hole sites (Fig. 5iv, viii). In contrast, IK1 labelling revealed a low level of membrane IK_Ca_ localization and discrete, punctate densities, which correspond to ∼77 and ∼23% of IEL holes in arteries from control and obese rats, respectively (Fig. 5i–iii and 5v–vii, respectively; Table S1).

**Figure 6:**
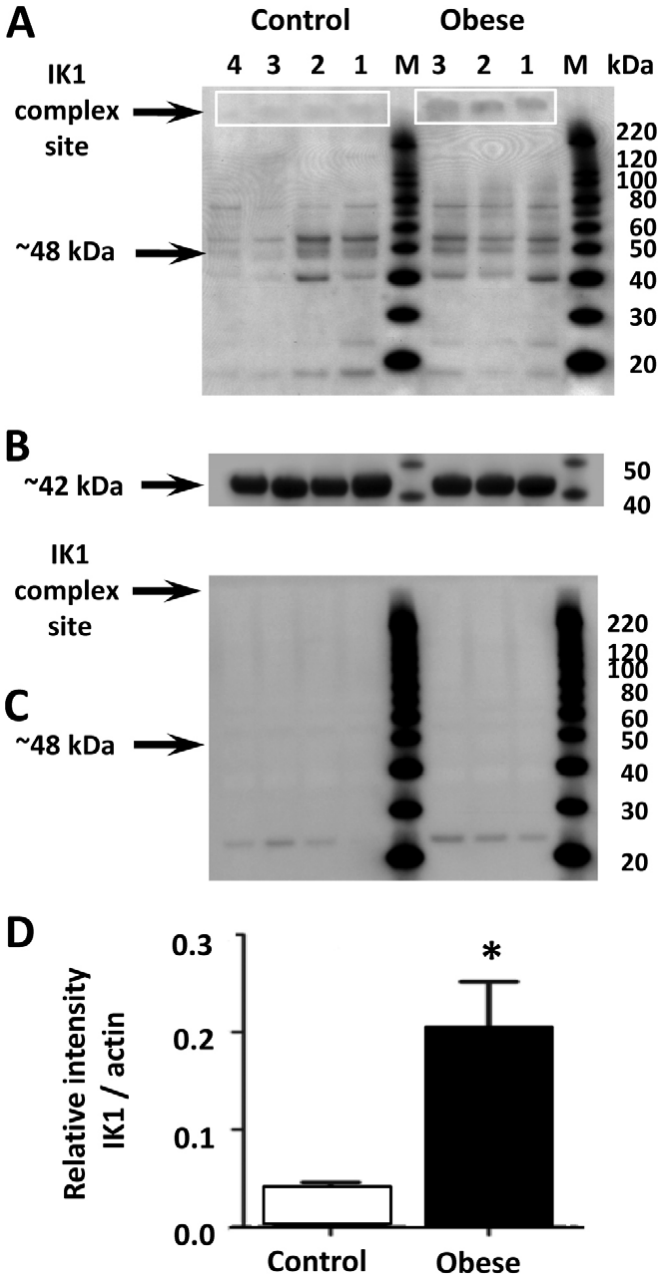
IK1 expression in control and obese rat mesenteric artery. Western blot data shows IK1 (IK_Ca_; **A–D**) expression in membrane extracts from control and obese rat mesenteric artery. High molecular weight SDS and heat resistant complexes are present (**A**) which have been demonstrated as the functional homotetrameric IK_Ca_ channels, whilst the monomeric ∼48 kDa IK_Ca_ protein is also present (**A**). Blots were stripped, reprobed with actin antibody and IK_Ca_ quantification normalized to β-actin expression (**B**); showing a significant increase in the relative intensity of IK_Ca_ (**D**) in obese rat arteries. As a control, antibody incubation in corresponding IK_Ca_ peptide shows absence of labeling (**C**). Lane M; molecular weight markers. Arrows indicate the position of the full length protein. *, significantly different from control (*P*<0.05; **D**).

In addition, IK1 (IK_Ca_) expression was determined using Western blotting (Fig. 6). Western blotting of IK_Ca_ using optimal selective antibodies (Tables S4 compared to S5) in the vasodilator field has the common problem that in general it is assumed that the molecular weight of the native single channels (∼48 kDa), reflects that of the functional channel (for example [14], [31], [32]). However, this is not the case, as high molecular weight complexes of these channels constitute the functional channel in both isolated cells and intact tissue [33], [34], [35], and thus, the expression of such complexes (and not the monomers) is of primary relevance for studies of functional K_Ca_ expression; and hence the reason for the focus on such complexes in the Western blotting of the present study. Further details of specific aspects of the IK_Ca_ quantification are included in Results S1.

Data show that IK_Ca_ were expressed in mesenteric arteries from control and obese animals (Fig. 6). IK1 appeared as a smeared band, characteristic of glycosylated and/or hydrophobic membrane proteins possessing multiple phosphorylation sites [36], [37]. Expression of the monomeric form of IK1 (∼48 kDa) did not differ between arteries of control and obese animals in either the soluble or membrane extracts (Fig. 6A). However, a high molecular weight IK1 complex that migrated at >220 kDa was present (Fig. 6A). This complex was more highly expressed in the membranes extracted from mesenteric arteries of obese animals (∼5.4-fold; *P<*0.05; Fig. 6A, D) and was absent from soluble extracts (data not shown).

The antibodies used in this study have been previously characterized using transfected cells and positive and negative controls, and shown to be fully selective [33], [34], [35], with peptide block of the primary antibodies abolishing staining of the bands corresponding to the IK1 proteins (Fig. 6C).

### Gap junction/connexin involvement in EDH

To clarify the potential functional role of heterocellular coupling in EDH activity in control and obese rat mesenteric arteries, membrane potential and vessel diameter were recorded in the presence of the putative gap junction uncoupler carbenoxelone (100 µM). In the presence of L-NAME (1 µM), ODQ (100 µM) and indomethacin (10 µM), carbenoxelone significantly reduced the ACh-mediated hyperpolarization in arteries from control and obese animals (*P<*0.05; Table S3). Carbenoxelone however, had no apparent effect on ACh-evoked relaxation in mesenteric arteries from control or obese animals (Table S2).

### K^+^ mediates EDH via decreased K_ir_ and increased Na^+^/K^+^-ATPase activation in obesity

**Figure 7:**
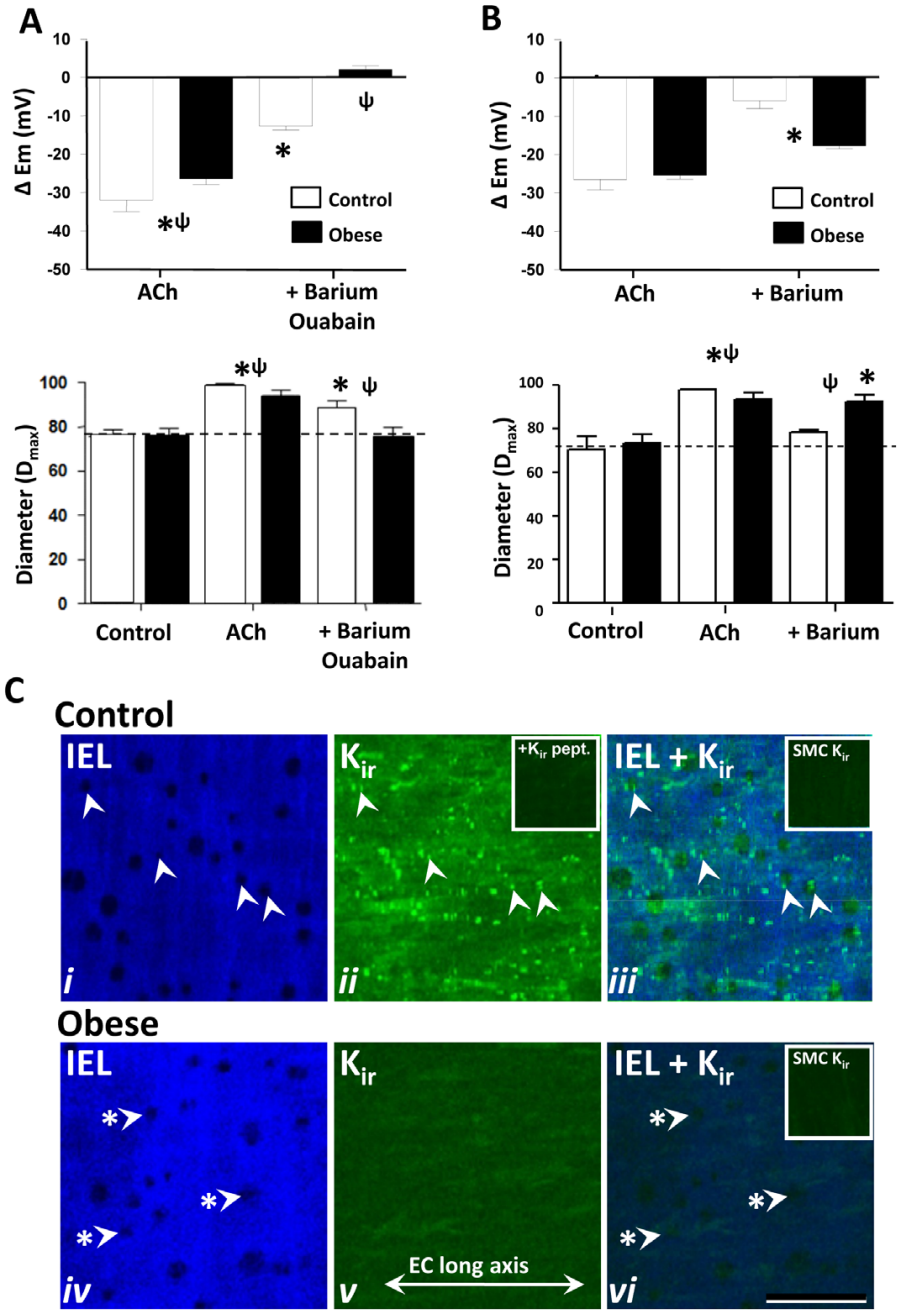
Na^**+**^/K^**+**^ATPase and K_ir_ activity, and K_ir_ distribution. Combined Na^+^/K^+^ATPase and K_ir_ inhibition with ouabain (100 µM) and barium (30 µM), respectively, attenuates ACh-mediated hyperpolarization and relaxation in control, and abolished the response in obese rat vessels (**A**). In arteries of control, barium alone reduced EDH activity, whilst in obese, barium alone had no effect on vessel diameter, although the ACh-evoked hyperpolarization was attenuated (**B**; Table S3). *P<0.05 from own control; ψ, *P*<0.05 control v obese. ΔE_m_, change in membrane potential from control. Confocal immunohistochemistry demonstrates K_ir_ distribution in control (**Ci–iii**) and obese (**Civ–vi**) rat arteries (same region shown in **i–iii** and **iv–vi**, respectively). Autofluorescence demonstrates internal elastic lamina (IEL) morphology and associated holes (**Ci, iv**, examples arrowed) as potential myoendothelial gap junction sites. In control arteries, K_ir_ labelling shows intense punctate localization at discrete points in the endothelium, including in close proximity to a proportion of IEL holes (**Cii–iii**, examples arrowed); an observation absent in obese rat vessels (**iv–vi**, arrows with asterisks indicate examples of such sites, **vi**). K_ir_ labelling was absent in the smooth muscle of both control and obese arteries (insets, **Ciii, vi**, respectively), whilst endothelial labelling in control was blocked by pre-incubation in K_ir_ antibody antigenic peptide (**Cii**, inset). Longitudinal vessel axis runs left to right (example, **v**). Bar, 20 µm.

The effect of dietary intervention on the selective characteristics of K_ir_- and Na^+^/K^+^-ATPase-dependent EDH was characterized in mesenteric artery of control and obese rats. Inhibition of K_ir_ and Na^+^/K^+^-ATPase by combined barium (30 µM) and ouabain (100 µM), respectively, reduced ACh-evoked EDH in arteries from control rats (*P<*0.05; Fig. 7A; Tables S2,S3), with barium alone causing a similar reduction in the magnitude of hyperpolarization and relaxation as combined K_ir_ and Na^+^/K^+^-ATPase block (*P*<0.05; Fig. 7B). In contrast, exposure to barium and ouabain in arteries from obese animals abolished ACh-evoked EDH (*P<*0.05), whilst barium alone caused a small, but significant reduction in ACh-induced EDH (*n = *4; *P<*0.05), with no significant effect on EDH-mediated relaxation (*n = *4; Fig. 7B; Tables S2,S3).

Confocal immunohistochemistry using characterized antibodies to examine the distribution of K_ir_ in arteries from control and obese rats revealed distinct punctate staining across the endothelial cell surface in arteries from control rats, with a low level of cell membrane/cytoplasmic K_ir_ labeling; both of which could not be detected in the endothelium of obese rat arteries (Fig. 7C). K_ir_ labeling was absent in the smooth muscle (Fig. 7Ciii, vi, insets). Like IK_Ca_, in control arteries, a proportion of localized K_ir_ densities corresponded with IEL holes, as potential myoendothelial microdomain/gap junction sites (Figs. 7Ci–iii, 8).

**Figure 8:**
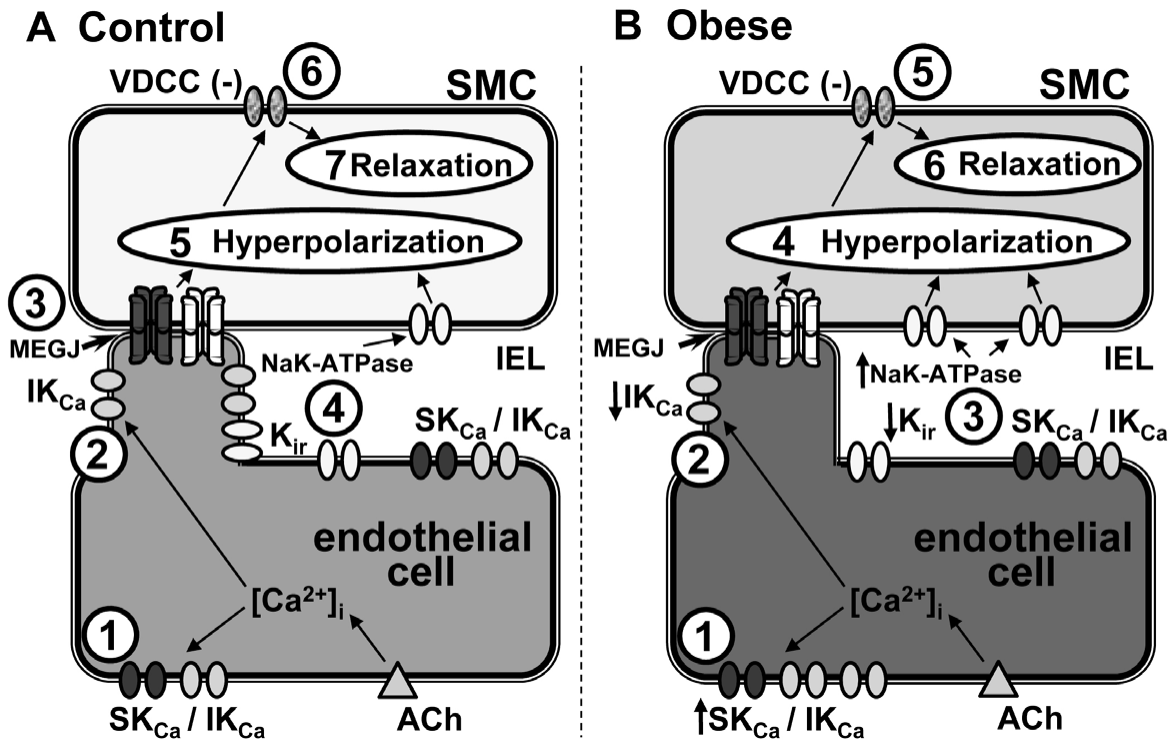
Model EDH mechanism. In arteries from control (**A**), agonist (ACh) elicits increased endothelial [Ca^2+^]_i_ and subsequent endothelial membrane SK_Ca_ and IK_Ca_ activation (1), including at IK_Ca_ densities at potential myoendothelial gap junction (MEGJ)-related microdomain signaling sites (2). The resulting endothelial hyperpolarization can spread to the smooth muscle via MEGJs (3). The hyperpolarization is amplified by smooth muscle Na^+^/K^+^-ATPase activation and endothelial K_ir_, following SK_Ca_ and IK_Ca_-mediated K^+^ efflux into the extracellular space (4), some of which are located at potential MEGJ-related signaling domains (**Fig. 5Cii, iii**; see also [13]). Smooth muscle hyperpolarization occurs (5), with closure of voltage-dependent calcium channels (6) and subsequent vessel relaxation (7). In arteries from obese animals (**B**), SK_Ca_ and IK_Ca_ are primarily located on the endothelial cell membrane (1); with IK_Ca_ incidence at MEGJ-related signaling domains being significantly decreased compared to control (2; **Table S1**). Since MEGJ incidence is unaltered, and the number of smooth muscle cell (SMC) layers increased in obesity compared to control, MEGJ-dependent EDH transfer *may* not be a sufficient driving force for smooth muscle relaxation. Under these circumstances, and in the absence of detectable K_ir_, EDH is primarily attributable to activation of smooth muscle Na^+^/K^+^-ATPase, following K^+^ efflux from primarily endothelial cell membrane SK_Ca_ and IK_Ca_ (3). Once sufficient hyperpolarization occurs (4), voltage-dependent calcium channels close (5) and relaxation results (6).

Use of Na^+^K^+^-ATPase α1 and α2 subunit-directed antibodies (Santa Cruz; sc-16043 and 31391, respectively) at serial dilutions of 1∶100–800 with confocal immunohistochemistry (in control artery only; *n = *3) produced inconclusive results. Antibody preincubation in a 10-fold excess of peptide corresponding to the sequences of these α1 and α2 antibodies only partially blocked apparent staining, and labeling was also present in endothelial cells with a similar intensity to that in the adjacent smooth muscle (suggesting non-specificity). Thus, further determination of Na^+^K^+^-ATPase distribution was not conducted. No difference in immunohistochemical antigen distribution was present with aldehyde compared to solvent fixation.

## Discussion

In the rat mesenteric artery, as a model bed for resistance vessel studies, impaired EDH activity underlies endothelial dysfunction associated with diet-induced obesity. In obese rat mesenteric artery, altered EDH is due to K^+^ release via upregulated endothelial IK_Ca_ and subsequent activation of smooth muscle Na^+^/K^+^-ATPase, whilst endothelial K_ir_ expression is reduced and myoendothelial gap junction density is unchanged compared to control. Increased IK_Ca_ expression and redistribution, and reduced SK_Ca_ and K_ir_ function, and altered distribution in arteries from obese rats, support the presence of plasticity in the potassium channel signaling mechanisms that underlie EDH in the mesenteric artery of obesity-related disease. Functional IK_Ca_ upregulation has been previously reported in mesenteric artery of stroke-prone spontaneously hypertensive rats, where EDH is impaired [38]. Indeed, alterations in both the functional contribution and expression of SK_Ca_ and IK_Ca_ are reported in mesenteric artery of diabetic obese Zucker, and in Sprague-Dawley rats during angiotensin II-induced hypertension [38], [39], where EDH is also impaired. Collectively, these data demonstrate the presence of similar alterations in the potassium channel mechanisms that underlie EDH in different models of vascular disease.

EDH dependence on SK_Ca_ and IK_Ca_ in the mesenteric artery of normal adult rats was confirmed by selective SK_Ca_ and IK_Ca_ block [3], [7], [40], and supported via direct endothelial SK_Ca_ and IK_Ca_ activation with CyPPA and 1-EBIO, and subsequent apamin/TRAM-34 block [16], [28], [29], [30], [41]. In mesenteric artery from obese rats, functional IK_Ca_ upregulation in arteries from obese rats is consistent with increased IK1 (IK_Ca_) expression and redistribution in such vessels; TRAM-34 reducing EDH to a greater extent in arteries of obese compared to control rats (by ∼93 *cf/.* 60%, respectively; Fig. 4C). Conversely, apamin reduced EDH to a greater extent in arteries of control compared to obese rats (by ∼70 *cf/.* 60%, respectively; Fig. 4A), supporting a reduced functional contribution of SK_Ca_ to EDH in obese. The lack of difference in CyPPA-induced EDH in control compared to obese may be due to its ability to sensitize SK_Ca_ to calcium [42], and a potential differential ability of vascular cells in disease states to buffer intracellular calcium, as previously described in obese and hypertensive rat models [43], [44].

In arteries from both control and obese rats, potential exists for the direct transfer of EDH from the endothelium to the smooth muscle via myoendothelial gap junctions (Figs. 1, 8), and such activity occurs in normal rat mesenteric artery [11], [12]. In contrast to the present study where myoendothelial gap junction density was the same in artery from control and obese rats, decreased myoendothelial gap junctional coupling was suggested to impair rat mesenteric artery EDH-type relaxation in Zucker obese insulin-resistant rats [15]; although quantification of such coupling was not undertaken in that study. However, in the Zucker, the underlying Cx-mediated mechanisms at such sites may be altered (for vascular Cx regulation review [45]), without a change in gap junction density. Furthermore, whilst medial hypertrophy occurs in the mesenteric artery of obese compared to control rats, similar to that observed in the rat saphenous artery during development [46], it had no apparent effect on EDH magnitude. Indeed, altered gap junctional coupling between adjacent vascular smooth muscle cells may be compromised in disease [45], resulting in a reduction in the apparent relative contribution of EDH to vasodilation in such states. Moreover, under disease conditions, such as diabetes, hypertension and obesity, structural changes in the vessel wall are typical [47], with diet-induced obese rat mesenteric artery having an increased media-to-lumen ratio, typical for resistance vessels in some vascular disease states [47], [48]. Since no difference in the passive diameters of control and obese rat mesenteric artery occurred, the present data are consistent with inward remodeling of obese rat mesenteric artery [48]. Additionally, consistent with these data, increased media-to-lumen ratio and medial thickness occur in small human subcutaneous resistance arteries in severe obesity where endothelium-dependent relaxation is impaired [49].

In this study, the putative gap junction uncoupler carbenoxelone was used in an attempt to assess the functional role of gap junctional coupling to EDH. Although this agent inhibits ACh-mediated EDH in mesenteric artery from control and obese rats, it had little effect on EDH-mediated relaxation; consistent with previous observations in rat mesenteric artery [13]. Since it has previously been shown that carbenoxelone prevents EDH by limiting the generation of EDH itself [50], it is unlikely that the reduced smooth muscle hyperpolarization observed in the present study is due to altered cellular coupling, but rather to non-gap junction channel, receptor and/or store effects of this inhibitor [50], [51]. Indeed, the specificity of other putative gap junction inhibitors such as the Cx-mimetic peptides and halothane has also been questioned [52], [53], ruling out their additional use in this study.

Individual aspects of the EDH mechanism may act synergistically or in parallel, via K^+^ efflux into the myoendothelial space, activating smooth muscle Na^+^K^+^-ATPase and endothelial K_ir_ to evoke membrane hyperpolarization, or via direct current transfer via myendothelial gap junctions (Fig. 8; [3], [5]); with potassium channel- and myoendothelial gap junction-mediated EDH mechanisms thus not necessarily being interdependent in the rat mesenteric artery. Indeed, myoendothelial contact sites are present in mesenteric and other arteries that do not have gap junction characteristics [54], [55], consistent with the possibility that such contact sites are where localized potassium release, but not necessarily gap junction mediated coupling occurs; with Table S2 data supporting this proposition (IEL hole myoendothelial gap junction and IK_Ca_ densities being different).

In arteries from control rats, ouabain and barium reduced ACh-mediated EDH by ∼50%, supporting the functional contribution of Na^+^K^+^-ATPase, K_ir_ and myoendothelial gap junctions in this artery, as previously suggested [13]. However, in the present study, barium and ouabain abolish ACh-mediated EDH in mesenteric arteries from obese animals. Together with the K_Ca_ data, this suggests that significant aspects of the EDH mechanism are altered in obesity. Since inhibition of K_ir_ with barium reduced EDH by a few mV without effect on the associated relaxation in obese rat artery, the small effect on smooth muscle membrane potential implies that some K_ir_ remain in such vessels; although the level is apparently below standard confocal immunohistochemistry protocol detection limits. Whilst the functional role of Na^+^K^+^-ATPase is considered in the present study, consideration of the anatomical role of these channels was limited by lack of reliable antibody. Interestingly, a reduction in endothelial K_ir_ function has been shown to underlie obesity-related endothelial dysfunction in arteries of human forearm [22].

In the present study, diet-induced obesity results in IK_Ca_ upregulation, which was mirrored by a ∼3-fold increase in IK1 (IK_Ca_) expression and concomitant reduction in SK_Ca_ function. Functional data suggest that an interaction of IK_Ca_ and Na^+^K^+^-ATPase is primarily responsible for EDH in mesenteric artery of obese rat. Furthermore, a differential spatial association of K_Ca_ and K_ir_ reflect functional changes in their contribution to EDH (Fig. 8). The present data suggest that restoration of endothelial potassium channel activity represents a potential selective target to correct endothelial vasodilator function in cardiovascular disease, such as that present in obesity. Future work will examine the nature of the EDH signaling mechanism in human arteries in health and disease, with a particular focus on clarifying whether the altered potassium channel signalling mechanism observed here is conserved between species, to confirm whether this mechanism is a valid target for therapeutic correction.

## Materials and Methods

### Animals, dietary intervention and biochemical parameters

All experiments were performed in accordance with the guidelines of the National Health and Medical Research Council of Australia and the Animal Experimentation Ethics Committees of the University of New South Wales (approval ID 09/43B) and the Australian National University.

Eight week old male Sprague Dawley rats were fed normal chow (control; ∼12% kJ as fat) or a cafeteria-style high fat diet (obese; ∼30% kJ as fat) for 16–20 weeks. The latter obese diet consisted of ground chow with commercially available condensed milk, meat pies, pasta, cakes and dims sims. The ∼30% fat content of this diet has previously been shown to result in characteristics typical of human dietary obesity, such as elevated blood pressure, glucose, insulin, leptin and triglycerides [24], [25]. Food was provided *ad lib* and body weight and food intake monitored weekly over the intervention period, with animals aged 24–28 weeks at termination. High-fat and control diets were available *ad lib* throughout, with non-fasted glucose, leptin and insulin, and retroperitoneal and gonadal fat and organ mass being measured immediately upon sacrifice. Glucose was measured in fresh blood samples taken via cardiac puncture (Accu-Chek Advantage, Australia, Castle Hill), with separated plasma being used to measure plasma insulin and leptin (Linco Research, USA).

### Isolated artery preparation

Rats were anaesthetized with thiopental sodium (100 mg/kg; i.p.). Mesenteric arteries (4^th^ order collateral branches) were isolated and placed into cold (5–7°C) dissection buffer containing (mM): 3 Mops; 1.2 NaH_2_PO_4_; 4.6 glucose; 2 pyruvate; 0.02 EDTA (Na); 0.15 albumin; 145 NaCl; 4.7 KCl; 2 CaCl_2_; 1.2 MgSO_4_. Artery segments were then cannulated with two glass micropipettes, secured using 10-0 nylon surgical sutures (Alcon, Australia) and mounted in a 3–5 ml recording chamber (Living Systems, USA). Arterial segments were superfused (3 ml min^−1^) with physiological salt solution (PSS; mM): 111 NaCl; 25.7 NaHCO_3_; 4.9 KCl; 2.5 CaCl_2_; 1.2 MgSO_4_; 1.2 KH_2_PO_4_; 11.5 glucose; 10 HEPES; gassed with 5% CO_2_ in nitrogen (37°C, pH 7.4); and pressurized under no flow conditions to 40 mmHg by connecting the inflow pipette to a pressure servo and peristaltic pump (Living Systems, USA; see [56]). Vessels were allowed to equilibrate for 40 min, following which, pressure steps in 10 mmHg increments from 40 to 80 mmHg were applied every 10 min. Spontaneous tone was typically seen to develop at the completion of this protocol. In order to obtain optimal physiological myogenic and drug responsiveness, the stability of the cannulation was tested at the onset of each experiment by transiently increasing luminal pressure to 110 mmHg. Only vessels without pressure leaks and displaying subsequent spontaneous myogenic tone were studied. The vessel diameter was continuously measured using video-microscopy (DIAMTRAK [57]). Drug solutions were superfused, except for 1-EBIO (300 µM) and CyPPA (30 µM) which were applied luminally, and allowed to equilibrate for at least 30 mins before responses were recorded. All recordings were made in the presence of indomethacin (10 µM), L-NAME (100 µM) and ODQ (10 µM) to inhibit prostaglandins, NO synthase and guanylyl cyclase, respectively. To obtain the maximum vessel diameter (D_max_), at the end of experiments, arteries were exposed to 0 mM Ca^2+^ PSS containing 2 mM EGTA.

Smooth muscle cells were impaled with sharp microelectrodes (120–185 MΩ) filled with propidium iodide (0.2% in 0.5 M KCl) to confirm cellular identity. Membrane potential recordings were amplified with an Axoclamp 900A (Molecular Devices, USA) and stored for analysis using pClamp software (v.10; Molecular Devices, USA). Successful recordings were characterized by an abrupt signal deflection upon cell impalement and an approximate return to pre-impalement values on removal of the microelectrode.

### Confocal immunohistochemistry

Rats were anaesthetized as described for isolated artery preparations and perfused with a clearing solution containing 0.1% NaNO_3_, 0.1% BSA and 10 U/ml heparin, followed by 2% paraformaldehyde in PBS for 10 min. Alternative fixation was also carried out on freshly isolated and dilated (0.1% NaNO_3_) vessels in cold acetone for 5 min. Mesenteric arteries, as above, were isolated, cut along the longitudinal plane and pinned out as a flat sheet, intima uppermost. Segments of artery from control and obese rats were then incubated in the same well, in blocking buffer containing 1% BSA, 0.2% Tween 20 for 2 h at room temperature, rinsed (3×5 min) in PBS and further incubated in primary antibody to SK3 (SK_Ca_; 1∶100, Chen, M75) and IK1 (IK_Ca_; 1∶100, Chen, M20), inwardly rectifying potassium channel (K_ir_; 1∶100, Santa Cruz, sc-18708), and Na^+^/K^+^ATPase (α1 and α2; 1∶100–800, Santa Cruz, sc-16043 and 39391, respectively) in blocking buffer for 18 h at 4°C. The tissue was again rinsed (3×5 min) and incubated in species-specific secondary antibody (Alexa Fluor 633; Invitrogen, Australia, A21070 and A21082, as appropriate), diluted in 0.01% Tween 20 for 2 h. Preparations were given a final 3×5 min rinse in PBS, mounted in anti-fade media and examined with a confocal microscope (Olympus FV1000) using uniform settings. Sequential images were recombined to create a single image incorporating all of the endothelial cell or smooth muscle cell labeling, above or below the focal plane of the internal elastic lamina (IEL), respectively. Individual sections within the IEL near the inner smooth muscle cell membrane were recombined with the same sections viewed at 488 nm excitation, to show IEL autofluorescence and potential myoendothelial microdomain sites, as previously described in this vessel [5], [10]. CellR software (Olympus) was used for quantitative measurements.

For K_Ca_, K_ir_ and Na^+^/K^+^ATPase antibodies, controls for specificity involved antibody peptide block, incubation without primary and absence of smooth muscle (K_Ca_/K_ir_) or endothelial (Na^+^/K^+^ATPase) staining in the same tissue sample; the latter effectively being an internal negative control. Additional K_Ca_ antibody controls previously conducted involved further positive and negative controls with transfected cells, Western blotting and immunoelectron microscopy [10], [33], [34], [52], [58]. Of note, this protocol has previously been shown to successfully label antigens expressed in smooth muscle cells [52], thus demonstrating that antibody access was not a limiting factor in the absence of labeling in this cell layer.

### Western blotting

Mesenteric arteries, as above, were dissected from age-matched control and obese rats, extraneous tissue was carefully removed and the arteries were stored in liquid nitrogen. Vessels from 4 animals per ‘*n*’ (*n* = 3 and 4, for each obese and control lane, respectively) were collected. The arteries were ground in liquid nitrogen using a pestle and mortar, resuspended in phosphate buffered saline (PBS) pH 7.4 containing complete protease inhibitor cocktail (Roche) and centrifuged (3000× g, 4°C, 5 min). The supernatant was removed and placed on ice and the pellet was snap frozen in liquid nitrogen and processed again as described above. Following the second spin the supernatants were pooled and centrifuged (25,000× g; 4°C; 1 h) and the supernatant, enriched in cytosolic proteins, was aliquoted, snap frozen in liquid nitrogen and stored at −80°C. The membrane-enriched pellet was carefully resuspended in PBS containing 0.1% Triton X-100 and protease inhibitor cocktail, aliquoted, snap frozen in liquid nitrogen and stored at −80°C. Protein concentration of the samples was determined using the Bradford protein assay (BioRad).

Aliquots of protein extracts (5 µg protein unless otherwise indicated) were dissolved in lithium dodecyl sulfate (LDS) sample buffer (0.5% LDS, 62.5 mM Tris-HCl, 2.5% glycerol, 0.125 mM EDTA, pH 8.5) for 10 min at 70°C. The samples were separated by electrophoresis in 4–12% bis-Tris polyacrylamide gels using MES SDS running buffer and electroblotted onto PVDF membranes overnight at 4°C, according to the manufacturer's recommendations (Invitrogen). Following transfer, blots were thoroughly washed, blocked, probed with primary antibody (Table S4 and S5) and specific binding was visualized using alkaline phosphatase-conjugated secondary antibody and chemiluminescence according to the manufacturer's instructions (Invitrogen). The intensity of the band corresponding to each protein was quantified by digital densitometry using ImageJ software (NIH). Relative intensity for each protein was determined by comparison with the intensity of actin staining on blots that were stripped and then reprobed with actin primary antibody (Fig. 6).

To determine specificity, each antibody was incubated with its cognate peptide in order to block specific binding. Prior to use, peptide was added to antibody in a 1∶1 ratio (w/w), mixed and incubated at 37°C for 1 h, then overnight at 4°C. The blocked antibody was then used in Western blotting detection as described above.

### Serial section transmission electron microscopy

Rats were anaesthetized as above and mesenteric arteries from control and diet-induced obese animals dissected after perfusion fixation (1% paraformaldehyde, 3% glutaraldehyde in 0.1 mM sodium cacodylate buffer with 10 mM betaine, pH 7.4), with short segments of artery being processed for electron microscopy [46]. Serial transverse sections (∼100 nm thick) were cut over ∼5 µm of vessel length. Myoendothelial gap junctions were identified according to their characteristic pentalaminar membrane structure, and together with their surrounding endothelial and smooth muscle regions were counted and imaged at ×10–40 k magnification at 16 megapixel resolution (camera from Scientific Instruments and Applications, Inc., Duluth, USA). Quantitative wall measurements were made from vessel cross-sections cut 90° perpendicular to the longitudinal vessel axis from ultrastructural montages taken at ×1.5–2.5 k at 16 megapixel resolution. Diameter was calculated from IEL circumference, and media to lumen ratio from medial thickness divided by lumen diameter. The number of smooth muscle cell layers was counted as the mean of cell profiles >5 µm long, 90° apart. CellR software was used for gross quantitative measurements.

### Drugs

Apamin, ACh, L-NAME, indomethacin, barium, ODQ and ouabain were obtained from Sigma-Aldrich (USA). TRAM-34, 1-EBIO was purchased from Toronto Research Chemicals (Canada) and Tocris (UK) and CyPPA obtained from NeuroSearch A/S (Denmark). All stock solutions were prepared in distilled water except for ODQ, 1-EBIO and TRAM-34 which were dissolved in DMSO, which was without effect at 100 µM.

### Analysis and statistics

Drug effects were determined by expressing the diameter as a percent of the maximum vessel diameter (% D_max_) recorded in 0 Ca^2+^ PSS with 2 mM EGTA. Results are given as a mean ± SEM of *n* rats. Statistical analysis was determined using 95% confidence limits (*P*<0.05), Student's *t* test for paired or unpaired data or one-way ANOVA, as indicated for specific protocols. Data analysis and graph production were performed using GraphPad Prism.

## Supporting Information

Results S1 Additional IK_Ca_ data.
(DOC)

Figure S1 Vessel wall morphology. Low magnification electron micrographs of vessel wall cross sections from control (A) and obese. (B) animals. For quantitative wall properties, see **Table 2**.
(PDF)

Table S1 Control and diet-induced obese rat mesenteric artery internal elastic lamina (IEL) hole, IK_Ca_ and myoendothelial gap junction (MEGJ) characteristics.
(DOC)

Table S2 Control and diet-induced obese rat mesenteric artery diameter characteristics and drug intervention.
(DOC)

Table S3 Control and diet induced obese rat mesenteric artery smooth muscle membrane potential (mV) characteristics and drug intervention.
(DOC)

Table S4 Western blot primary antibody characteristics.
(DOC)

Table S5 Additional Western blot primary antibody characteristics.
(DOC)
