# Supplementary material for: Diet-Induced Obesity Impairs Endothelium-Derived Hyperpolarization via Altered Potassium Channel Signaling Mechanisms
Source: PLoS One. 2011 Jan 21;6(1):e16423. doi: 10.1371/journal.pone.0016423 (PMC3025034; doi:10.1371/journal.pone.0016423)
Supplement: Results S1 — Additional IKCa data. (DOC) [file pone.0016423.s001.doc]

**Supporting Information**

**Results**

*Additional IK_Ca_ data*

The IK1 M20 antibody used was characterized by Western blotting and immunolabeling of transfected cells, and have been demonstrated to be highly selective in several independent studies (Table S4 [1,2,3]). Furthermore, it has been demonstrated that some potassium channels, including IK_Ca_ (as IK1 / SK4 / KCa3.1 / KCNN4), when assembled as homotetrameric complexes are resistant to dissociation by heat and detergents and migrate in SDS-PAGE gels as smeared complexes [1,2,3,4,5]. Exhaustive and independent studies by Boettger *et al.* [2], Chen *et al*. [1] and Mongan *et al.* [3] characterize a series of antibodies recognising each K_Ca_ subtype, and show that IK_Ca_ appears as a series of smeared bands migrating between ~50 and >250 kDa (see also Fig. 4C in Absi *et al.* [6]). This is also the case in the present study (Fig. 6A), which was carried out under mild conditions for the extraction and electrophoresis of membrane proteins from whole arteries. The high molecular weight complex >220 kDa represents the functional homotetrameric channel complex and we therefore used this to quantify IK_Ca_ protein expression. Lower molecular weight bands represent a combination of degradation products or trimers, dimers, and monomers, and which therefore do not represent the functional homotetrameric channel complex [1,2]. Peptide block of the primary antibody abolished staining that corresponded to specific IK_Ca_ protein (Fig. 6C), with bands and block reflecting that present in cultured IK_Ca_-transfected cells [1,2,3]. Thus, simply stated, antibody specificity in the present study is reflected by mimic of the specific location and block of bands in IK_Ca_ transfected cells [1,2,3]. Additional IK_Ca_ targeted antibodies were also used to probe Western blots, but were found to not optimally identify bands at predicted molecular weights, and / or had significant background (Table S5). The apparent faint appearance of the specific IK_Ca_ bands (Fig. 6A) is likely a reflection of the titre and affinity of these antibodies for the IK_Ca_ membrane proteins in the mesenteric artery and an indication that the proteins are not highly abundant in the samples which included all membrane material extracted from whole arteries consisting of endothelium, smooth muscle and connective tissue.

It is important to note that the samples for Western blotting are not exclusively composed of plasma membranes and will contain other cellular organelles including mitochondria, lysosomes, peroxisomes, and possibly golgi and endoplasmic reticulum, which may affect the interpretation of the changes in IK_Ca_ expression in the present study, particularly as IK_Ca_ expression has been described in mitochondria [7].

References

1. Chen MX, Gorman SA, Benson B, Singh K, Hieble JP, et al. (2004) Small and intermediate conductance Ca^2+^-activated K^+^ channels confer distinctive patterns of distribution in human tissues and differential cellular localisation in the colon and corpus cavernosum. Naunyn Schmiedebergs ArchPharmacol 369: 602-615.

2. Boettger MK, Till S, Chen MX, Anand U, Otto WR, et al. (2002) Calcium-activated potassium channel SK1- and IK1-like immunoreactivity in injured human sensory neurones and its regulation by neurotrophic factors. Brain 125: 252-263.

3. Mongan LC, Hill MJ, Chen MX, Tate SN, Collins SD, et al. (2005) The distribution of small and intermediate conductance calcium-activated potassium channels in the rat sensory nervous system. Neuroscience 131: 161-175.

4. Arkin IT, Sukharev SI, Blount P, Kung C, Brunger AT (1998) Helicity, membrane incorporation, orientation and thermal stability of the large conductance mechanosensitive ion channel from *E. coli.* Biochim Biophys Acta 1369: 131-140.

5. Corey S, Clapham DE (1998) Identification of native atrial G-protein-regulated inwardly rectifying K^+^ (GIRK4) channel homomultimers. J Biol Chem 273: 27499-27504.

6. Absi M, Burnham MP, Weston AH, Harno E, Rogers M, et al. (2007) Effects of methyl beta-cyclodextrin on EDHF responses in pig and rat arteries; association between SK(Ca) channels and caveolin-rich domains. Br J Pharmacol 151: 332-340.

7. Sassi N, De Marchi U, Fioretti B, Biasutto L, Gulbins E, et al. (2010) An investigation of the occurrence and properties of the mitochondrial intermediate-conductance Ca^2+^-activated K^+^ channel mtK_Ca_3.1. Biochim Biophys Acta 1797: 1260-1267.
