# Supplementary material for: Diet-Induced Obesity Impairs Endothelium-Derived Hyperpolarization via Altered Potassium Channel Signaling Mechanisms
Source: PLoS One. 2011 Jan 21;6(1):e16423. doi: 10.1371/journal.pone.0016423 (PMC3025034; doi:10.1371/journal.pone.0016423)

Figure S1. Vessel wall morphology.
Low magnification electron micrographs of vessel wall cross sections from control (A) and obese (B) animals. For quantitative wall properties, see **Table 2**.

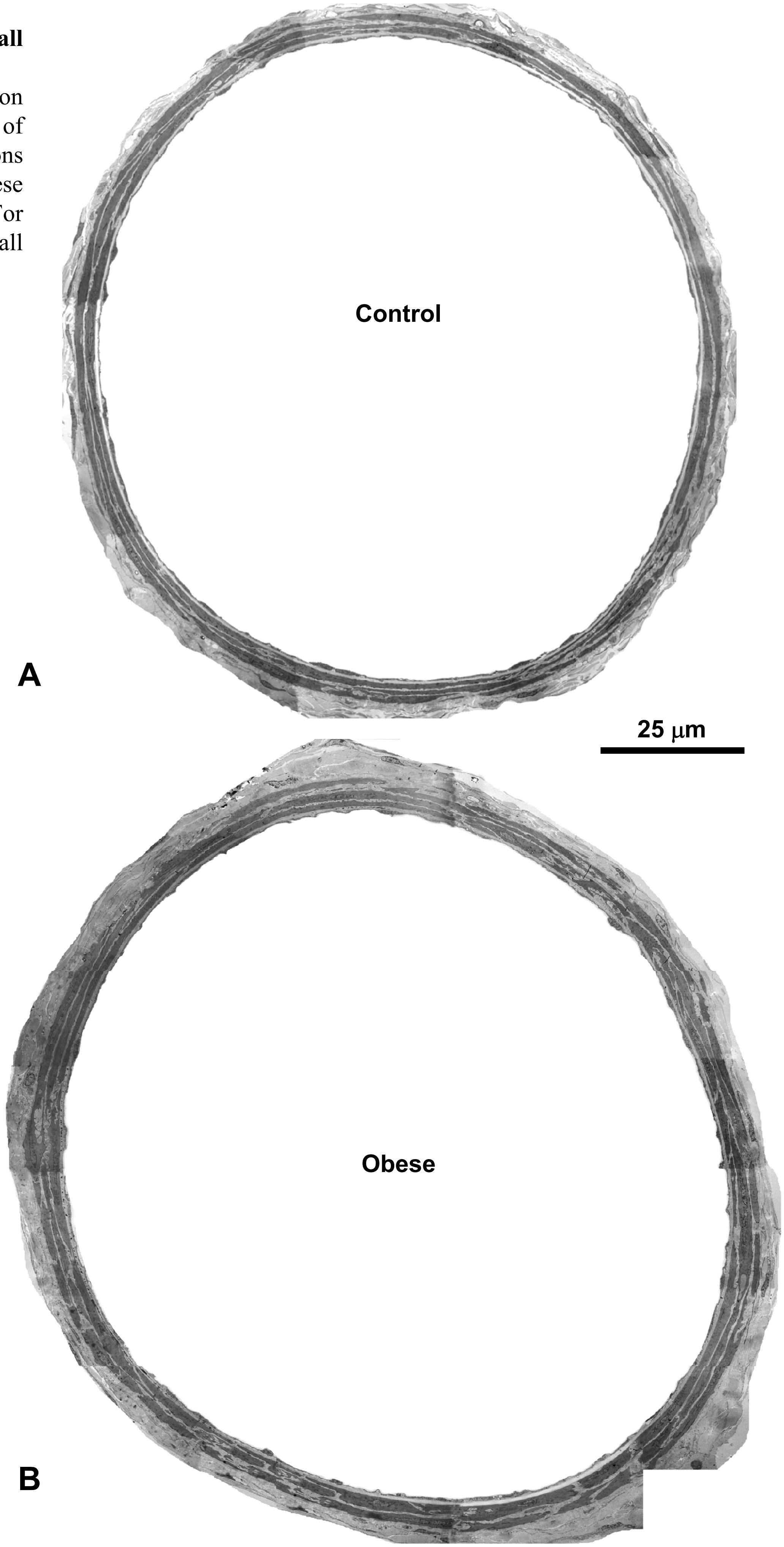

Supplement: Figure S1 — Vessel wall morphology. Low magnification electron micrographs of vessel wall cross sections from control (A) and obese. (B) animals. For quantitative wall properties, see Table 2 . (PDF) [file pone.0016423.s002.pdf]
