# Supplementary material for: Diet-Induced Obesity Impairs Endothelium-Derived Hyperpolarization via Altered Potassium Channel Signaling Mechanisms
Source: PLoS One. 2011 Jan 21;6(1):e16423. doi: 10.1371/journal.pone.0016423 (PMC3025034; doi:10.1371/journal.pone.0016423)
Supplement: Table S1 — Control and diet-induced obese rat mesenteric artery internal elastic lamina (IEL) hole, IKCa and myoendothelial gap junction (MEGJ) characteristics. (DOC) [file pone.0016423.s003.doc]

**Supporting Information**

**Table S1. Control and diet-induced obese rat mesenteric artery internal elastic lamina (IEL) hole, IK_Ca_ and myoendothelial gap junction (MEGJ) characteristics.**

|  | **A** | | | **B** | **C** | | **D** | | **E** | |
| --- | --- | --- | --- | --- | --- | --- | --- | --- | --- | --- |
|  | IEL holes /  10^3^ µm^2^ | | | ^1.^IK1 plaques at IEL holes / 10^3^ µm^2^ | ^2.^IK1 plaques not at IEL holes / 10^3^ µm^2^ | | IK1 plaque size (µm^2^) | | MEGJs /  10^3^ μm^2^ | |
| Control | | 29 ± 2.6 | 22.3 ± 3.2;  ~77% of IEL holes | | 1.5 ± 0.5;  ~8% of IK1 plaques | 11.7 ± 2.5 | | 4.2 ± 0.6;  ~15% of IEL holes | |  |
| Obese | | 22 ± 2.9 | 5 ± 1*;  ~23% of IEL holes | | 21.0 ± 4.6*;  ~70% of IK1 plaques | 3.8 ± 0.7* | | 3.9 ± 0.4;  ~18% of IEL holes | |  |

*n=*4, each from a different rat; each *n* being the mean of four different randomly selected 2 x 10^3^ μm^2^ (**A-D**), and the number of MEGJs in 50 serial sections (**E**). *, *P*<0.05, compared to control. 1. Plaques located at and 2. not at internal elastic lamina (IEL) holes are defined as those within ≤2 µm and >2 µm of an IEL hole, respectively. Note that multiple IK1 plaques within 2 μm of each other were counted as a single IK1 plaque.
