# Supplementary material for: Diet-Induced Obesity Impairs Endothelium-Derived Hyperpolarization via Altered Potassium Channel Signaling Mechanisms
Source: PLoS One. 2011 Jan 21;6(1):e16423. doi: 10.1371/journal.pone.0016423 (PMC3025034; doi:10.1371/journal.pone.0016423)
Supplement: Table S2 — Control and diet-induced obese rat mesenteric artery diameter characteristics and drug intervention. (DOC) [file pone.0016423.s004.doc]

**Supporting Information**

**Table S2.** Control and diet-induced obese rat mesenteric artery diameter characteristics and drug intervention.

|  | **Control** | ***n*** | **Obese** | ***n*** |
| --- | --- | --- | --- | --- |
| diameter (µm)  0 mM Ca^2+^ PSS (D_max_) | 321.6 ± 8.6 | 53 | 325.9 ± 7.8 | 58 |
| Myogenic tone at 80 mmHg (%D_max_) | 74.1 ± 2.1 | 53 | 68.6 ± 2.5 | 58 |
| ACh (1 µM) + L-NAME (1 µM) + ODQ (10 µM) + indo (10 µM) | 96.6 ± 1.2 | 17 | 89.5 ± 4.5* | 23 |
|  |  |  |  |  |
| myogenic tone | 63.7 ± 8.2 | 5 | 77.2 ± 4.8 | 5 |
| ACh (1 µM) alone^#^ | 97.6 ± 1.1 | 5 | 95.9 ± 1.0* | 5 |
| +L-NAME (1 µM) + ODQ (10 µM)  + indo (10 µM) | 97.8 ± 1.2 | 5 | 93.3 ± 3.1* | 5 |
|  |  |  |  |  |
| myogenic tone | 76.8 ± 5.1 | 4 | 78.8 ± 4.8 | 4 |
| Apamin (50 nM) + TRAM-34 (1 µM) | 75.1 ± 3.9 | 4 | 70.3 ± 3.7 | 4 |
|  |  |  |  |  |
|  | **Control (% Dmax)** | ***n*** | **Obese (% Dmax)** | ***n*** |
| myogenic tone | 74.2 ± 2.7 | 4 | 74.6 ± 3.4 | 4 |
| ACh (1 µM) | 98.4 ± 0.6 | 4 | 93.1 ± 1.4* | 4 |
| + apamin (50 nM) + TRAM-34 (1 µM) | 73.8 ± 4.6 | 4 | 65.0 ± 6.9 | 4 |
|  |  |  |  |  |
| myogenic tone | 79.0 ± 3.0 | 4 | 73.4 ± 4.5 | 4 |
| CyPPA (30 µM) + 1-EBIO (300 µM) | 97.2 ± 1.3 | 4 | 95.3 ± 1.8 | 4 |
| + apamin (50 nM) + TRAM-34 (1 µM) | 74.0 ± 5.6 | 4 | 64.0 ± 7.5 | 4 |
|  |  |  |  |  |
| myogenic tone | 75.3 ± 7.0 | 5 | 72.8 ± 4.6 | 11 |
| ACh (1 µM) | 99.2 ± 0.5 | 5 | 95.9 ± 1.8* | 11 |
| + apamin (50 nM) | 75.1 ± 7.8 | 5 | 81.5 ± 5.3 | 11 |
|  |  |  |  |  |
| myogenic tone | 75.7 ± 3.6 | 9 | 76.4 ± 4.1 | 4 |
| CyPPA (30 µM) | 90.0 ± 1.2 | 9 | 92.0 ± 3.9 | 4 |
| + apamin (50 nM) | 72.9 ± 5.2 | 9 | 70.4 ± 7.9 | 4 |
|  |  |  |  |  |
| myogenic tone | 65.4 ± 8.8 | 5 | 60.5 ± 3.1 | 6 |
| ACh (1 µM) | 97.8 ± 0.9 | 5 | 84.7 ± 7.5* | 6 |
| + TRAM-34 (1 µM) | 79.9 ± 8.9 | 5 | 55.9 ± 2.4* | 6 |
|  |  |  |  |  |
| myogenic tone | 77.1 ± 0.8 | 4 | 69.0 ± 6.3 | 4 |
| 1-EBIO (300 µM) | 86.4 ± 1.3 | 4 | 91.5 ± 2.8* | 4 |
| + TRAM-34 (1 µM) | 77.5 ± 7.8 | 4 | 53.7 ± 6.8* | 4 |
|  |  |  |  |  |
| myogenic tone | 73.4 ± 6.4 | 4 | 74.0 ± 3.6 | 4 |
| ACh (1 µM) | 91.7 ± 1.2 | 4 | 95.2 ± 4.1 | 4 |
| + carbenoxelone (100 µM) | 90.3 ± 3.2 | 4 | 96.7 ± 0.3* | 4 |
|  |  |  |  |  |
| myogenic tone | 76.1 ± 3.0 | 4 | 76.2 ± 4.1 | 4 |
| ACh (1 µM) | 99.4 ± 0.3 | 4 | 95.6 ± 3.3* | 4 |
| + barium (30 µM) + ouabain (100 µM) | 89.9 ± 3.1 | 4 | 75.5 ± 5.0* | 4 |
|  |  |  |  |  |
| myogenic tone | 73.8 ± 6.9 | 4 | 74.3 ± 5.7 | 4 |
| ACh (1 µM) | 97.4 ± 0.7 | 4 | 94.7 ± 3.3* | 4 |
| + barium (30 µM) | 77.1 ± 5.6 | 4 | 92.4 ± 2.7* | 4 |

*^#^NB. Unless stated otherwise, all data recorded in the presence of L-NAME (100 µM), ODQ (10 µM) and indomethacin (10 µM).* ACh, acetylcholine; CBX, carbenoxolone; CyPPA, cyclohexyl-[2-(3,5-dimethyl-pyrazol-1-yl)-6-methyl-pyrimidin-4-yl]-amine; 1-EBIO, 1-ethyl-2-benzimidazolinone; indo, indomethacin; L-N (L-NAME), *N*_ω_-Nitro-L-arginine methyl ester hydrochloride; ODQ, 1H-[1,2,4]oxadiazolo[4,3-a]quinoxalin-1-one; TRAM-34, 1-[(2-chlorophenyl)diphenyl-methyl]-1H pyrazole. *, *P<*0.05, compared to control.
