# Supplementary material for: Diet-Induced Obesity Impairs Endothelium-Derived Hyperpolarization via Altered Potassium Channel Signaling Mechanisms
Source: PLoS One. 2011 Jan 21;6(1):e16423. doi: 10.1371/journal.pone.0016423 (PMC3025034; doi:10.1371/journal.pone.0016423)
Supplement: Table S3 — Control and diet induced obese rat mesenteric artery smooth muscle membrane potential (mV) characteristics and drug intervention. (DOC) [file pone.0016423.s005.doc]

**Supporting Information**

**Table S3.** Control and diet induced obese rat mesenteric artery smooth muscle membrane potential (mV) characteristics and drug intervention.

|  | **Control (mV)** | ***n*** | **Obese (mV)** | ***n*** |
| --- | --- | --- | --- | --- |
| Resting membrane potential (RMP) at 80 mmHg | -41.5 ± 0.5 | 53 | -41.6 ± 0.4 | 58 |
| ACh (1 µM) + L-NAME (1 µM) + ODQ (10 µM) + indo (10 µM) | -74.1 ± 2.1 | 53 | -68.6 ± 1.3 | 58 |

|  | **Control (mV)** | ***n*** | **Obese (mV)** | ***n*** |
| --- | --- | --- | --- | --- |
| RMP | -43.3 ± 0.6 | 4 | -42.5 ± 1.0 | 4 |
| ACh (1 µM) | -76.5 ± 1.4 | 4 | -70.5 ± 0.5* | 4 |
| + apamin (50 nM) + TRAM-34 (1 µM) | -43.7 ± 0.8 | 4 | -41.9 ± 1.0 | 4 |
|  |  |  |  |  |
| RMP | -41.7 ± 2.0 | 4 | -39.7 ± 1.4 | 4 |
| CyPPA (30 µM) + 1-EBIO (300 µM) | -74.5 ± 4.9 | 4 | -72.2 ± 0.6 | 4 |
| + apamin (50 nM) + TRAM-34 (1 µM) | -42.7 ± 0.2 | 4 | -40.7 ± 1.6 | 4 |
|  |  |  |  |  |
| RMP | -38.5 ± 0.5 | 5 | -42.2 ± 0.8 | 11 |
| ACh (1 µM) | -74.5 ± 2.2 | 5 | -68.4 ± 1.2* | 11 |
| + apamin (50 nM) | -50 ± 1.9 | 5 | -53.5 ± 2.4 | 11 |
|  |  |  |  |  |
| RMP | -42.0 ± 0.5 | 9 | -41.9 ± 1.5 | 4 |
| CyPPA (30 µM) | -58.3 ± 1.9 | 9 | -60.8 ± 0.8 | 4 |
| + apamin (50 nM) | -43.5 ± 0.9 | 9 | -41.6 ± 1.2 | 4 |
|  |  |  |  |  |
| RMP | -41.9 ± 2.5 | 5 | -40.8 ± 1.1 | 6 |
| ACh (1 µM) | -73.7 ± 1.8 | 5 | -69.0 ± 1.4* | 6 |
| + TRAM-34 (1 µM) | -54.5 ± 2.5 | 5 | -42.7 ± 0.8* | 6 |
|  |  |  |  |  |
| RMP | -41.2 ± 0.9 | 4 | -42.2 ± 0.5 | 4 |
| 1-EBIO (300 µM) | -57.8 ± 0.4 | 4 | -63.8 ± 1.0* | 4 |
| + TRAM-34 (1 µM) | -41.9 ± 0.6 | 4 | -38.7 ± 1.5 | 4 |
|  |  |  |  |  |
| RMP | -41.0 ± 2.0 | 4 | -41.0 ± 1.1 | 4 |
| ACh (1 µM) | -73.2 ± 2.2 | 4 | -68.3 ± 2.2* | 4 |
| + carbenoxolone (100 µM) | -49.1 ± 4.7 | 4 | -53.8 ± 2.6 | 4 |
|  |  |  |  |  |
| RMP | -41.0 ± 2.0 | 4 | -41.0 ± 1.1 | 4 |
| ACh (1 µM) | -73.9 ± 2.8 | 4 | -68.3 ± 1.5* | 4 |
| + barium (30 µM) + ouabain (100 µM) | -53.2 ± 2.0 | 4 | -39.7 ± 1.2* | 4 |
|  |  |  |  |  |
| RMP | -41.9 ± 0.4 | 4 | -41.2 ± 0.6 | 4 |
| ACh (1 µM) | -68.5 ± 2.9 | 4 | -66.6 ± 0.8 | 4 |
| + barium (30 µM) | -48.3 ± 1.6 | 4 | -60.7 ± 1.2* | 4 |

*^#^NB. Unless stated otherwise, all data recorded in the presence of L-NAME (100 µM), ODQ (10 µM) and indomethacin (10 µM).* ACh, acetylcholine; CBX, carbenoxolone; CyPPA, cyclohexyl-[2-(3,5-dimethyl-pyrazol-1-yl)-6-methyl-pyrimidin-4-yl]-amine; 1-EBIO, 1-ethyl-2-benzimidazolinone; indo, indomethacin; L-N (L-NAME), *N*_ω_-Nitro-L-arginine methyl ester hydrochloride; ODQ, 1H-[1,2,4]oxadiazolo[4,3-a]quinoxalin-1-one; TRAM-34, 1-[(2-chlorophenyl)diphenyl-methyl]-1H pyrazole. *, *P<*0.05, compared to control.
