# Supplementary material for: Diet-Induced Obesity Impairs Endothelium-Derived Hyperpolarization via Altered Potassium Channel Signaling Mechanisms
Source: PLoS One. 2011 Jan 21;6(1):e16423. doi: 10.1371/journal.pone.0016423 (PMC3025034; doi:10.1371/journal.pone.0016423)
Supplement: Table S4 — Western blot primary antibody characteristics. (DOC) [file pone.0016423.s006.doc]

**Supporting Information**

**Table S4.** Western blot primary antibody characteristics.

| Antigen | Host | Antibody | Source & Identifier | Dilution |
| --- | --- | --- | --- | --- |
| IK_Ca_,^#^ hu N’ aa 2-17 | Rabbit | Affinity purified polyclonal | M20 [1] | 1:250 |
| Actin | Rabbit | Affinity purified polyclonal | Sigma, A2066 | 1:1000 |
| Goat IgG  (Fc portion) | Rabbit | Affinity purified polyclonal AP conjugate | Invitrogen, WB7108 | n/a |

#, as IK1 / SK4 / K_Ca_3.1 / KCNN1. aa, amino acid; AP, alkaline phosphatase; hu, human; n/a, not indicated by manufacturer. IK_Ca_ batch M20 was from Mark Chen (GSK, Stevenage, UK).

References

1. Chen MX, Gorman SA, Benson B, Singh K, Hieble JP, et al. (2004) Small and intermediate conductance Ca^2+^-activated K^+^ channels confer distinctive patterns of distribution in human tissues and differential cellular localisation in the colon and corpus cavernosum. Naunyn Schmiedebergs Arch Pharmacol 369: 602-615.
