# Supplementary material for: Diet-Induced Obesity Impairs Endothelium-Derived Hyperpolarization via Altered Potassium Channel Signaling Mechanisms
Source: PLoS One. 2011 Jan 21;6(1):e16423. doi: 10.1371/journal.pone.0016423 (PMC3025034; doi:10.1371/journal.pone.0016423)
Supplement: Table S5 — Additional Western blot primary antibody characteristics. (DOC) [file pone.0016423.s007.doc]

**Supporting Information**

**Table S5. Additional Western blot primary antibody characteristics.**

| Antigen | Host | Antibody | Source & Identifier | Dilution |
| --- | --- | --- | --- | --- |
| IK_Ca_,^#^ hu N’ aa 2-17 | Rabbit | Affinity purified polyclonal | M4 [1] | 1:500 |
| IK_Ca_,^#^ rat C’ aa 350-363 | Rabbit | Affinity purified polyclonal | Alomone APC-064 (batch AN-02) | 1:500 |
| IK_Ca_,^#^ rat C’ aa 350-363 | Rabbit | Affinity purified polyclonal | Sigma P4997 (batch 03K1673) | 1:500 |

#, as IK1 / SK4 / K_Ca_3.1 / KCNN1. aa, amino acid. hu, human. IK_Ca_ batch M4 was from Mark Chen (GSK, Stevenage, UK).

References

1. Chen MX, Gorman SA, Benson B, Singh K, Hieble JP, et al. (2004) Small and intermediate conductance Ca^2+^-activated K^+^ channels confer distinctive patterns of distribution in human tissues and differential cellular localisation in the colon and corpus cavernosum. Naunyn Schmiedebergs Arch Pharmacol 369: 602-615.
